# Innate immune responses induced by the saponin adjuvant Matrix-M in specific pathogen free pigs

**DOI:** 10.1186/s13567-017-0437-2

**Published:** 2017-05-22

**Authors:** Viktor Ahlberg, Bernt Hjertner, Per Wallgren, Stina Hellman, Karin Lövgren Bengtsson, Caroline Fossum

**Affiliations:** 1 Department of Biomedical Sciences and Veterinary Public Health, Swedish University of Agricultural Sciences, SLU, Uppsala, Sweden; 2 National Veterinary Institute, SVA, Uppsala, Sweden; 3 Novavax AB, Uppsala, Sweden

## Abstract

Saponin-based adjuvants have been widely used to enhance humoral and cellular immune responses in many species, but their mode of action is not fully understood. A characterization of the porcine transcriptional response to Matrix-M was performed in vitro using lymphocytes, monocytes or monocyte-derived dendritic cells (MoDCs) and in vivo. The effect of Matrix-M was also evaluated in specific pathogen free (SPF) pigs exposed to conventionally reared pigs. The pro-inflammatory cytokine genes *IL1B* and *CXCL8* were up-regulated in monocytes and lymphocytes after Matrix-M exposure. Matrix-M also induced *IL12B*, *IL17A* and *IFNG* in lymphocytes and IFN-α gene expression in MoDCs. Several genes were indicated as up-regulated by Matrix-M in blood 18 h after injection, of which the genes for IFN-α and TLR2 could be statistically confirmed. Respiratory disease developed in all SPF pigs mixed with conventional pigs within 1–3 days. Two out of four SPF pigs injected with saline prior to contact exposure displayed systemic symptoms that was not recorded for the four pigs administered Matrix-M. Granulocyte counts, serum amyloid A levels and transcription of *IL18* and *TLR2* coincided with disease progression in the pigs. These results support further evaluation of Matrix-M as a possible enhancer of innate immune responses during critical moments in pig management.

**Electronic supplementary material:**

The online version of this article (doi:10.1186/s13567-017-0437-2) contains supplementary material, which is available to authorized users.

## Introduction

Immunostimulatory effects of saponins from *Quillaja saponaria* Molina are known since nearly 100 years. These saponins have on numerous occasions been used in veterinary vaccines, but the mechanisms underlying the effects are not fully understood [1–4]. Purified fractions of *Quillaja* saponins combined with phospholipids and cholesterol can be formulated into nanoparticle ISCOM-Matrix adjuvants [5, 6], such as the Matrix-M™ (Novavax AB, Uppsala, Sweden). Immunization experiments using Matrix-M as adjuvant reveal an antigen specific immune response characterized by long-lasting antibody production, a balanced T_H_1/T_H_2 cytokine profile and induction of cytotoxic T cells [7–9]. These adjuvant properties of Matrix-M also seem to increase the otherwise poor immunogenicity of Ebola virus glycoproteins [10].

In the pig, experimental vaccines formulated with ISCOM-Matrix have provided protection against pseudorabies [11], rotavirus [12, 13], *Toxoplasma gondii* [14] and *Mycoplasma hyopneumoniae* [15]. Intramuscular injection of the adjuvant component Matrix-M alone in pigs resulted in cellular influx to the draining lymph node [16] as also seen for neutrophils, dendritic cells, monocytes and NK cells after subcutaneous injection of Matrix-M in mice [17, 18] and for monocytes in calves [19]. The innate immune reaction to intramuscular injection of Matrix-M in pigs was further characterized by a type I interferon-related transcriptional response both at the site of administration and in the draining lymph node [16, 20]. These indications that Matrix-M seems to activate the interferon system, amongst other innate immune parameters, raises the possibility for its strategic use in pigs that are extra vulnerable to infections.

In modern pig husbandry disease susceptibility has been associated with weaning, transport and mixing, especially of pigs with different health status [21]. Physiological stress and infectious agents co-operate and preventive treatments include administration of IFN-α [22, 23] and β-glucans [24, 25]. Immunomodulators as a tool to boost the immune system against possible infections can potentially also be used in emergency vaccines [26]. In recent years it has been proposed that vaccine induced short-term innate immune responses possess a type of memory manifesting itself in a prolonged activation of innate immune cells which also acts on heterologous targets [27]. These non-specific effects of vaccines are now often referred to as “trained innate immunity” implying that priming of monocyte-derived cells and NK-cells by vaccination affects their future response to non-related agents [28].

The aim of the present study was to expand our understanding of how the saponin adjuvant Matrix-M may affect the response to different stressors such as infection and/or transport, building on previously acquired results on effects of Matrix-M administration to the pig [16, 20]. Therefore, we further evaluated modulatory effects of Matrix-M on innate immune parameters in the pig. To accomplish this, the host response to Matrix-M was evaluated in a 6-day contact exposure model using specific pathogen free (SPF) pigs treated with Matrix-M or saline 1 day before transport and mixing with conventionally reared pigs. During this period leukocyte counts, serum amyloid A (SAA) and expression of immune related genes were monitored in blood. Furthermore, gene expression results from blood were complemented by those from in vitro exposure of porcine peripheral blood mononuclear cell (PBMC) subpopulations to Matrix-M.

## Materials and methods

### Animals

The SPF pigs (Yorkshire × Landrace) originated from a herd (Serogrisen, Ransta, Sweden) declared free from major swine pathogens. The conventionally reared pigs came from a farrow-to-finish herd with a high prevalence of respiratory lesions recorded at slaughter. Pigs in this herd were in general seronegative to *Actinobacillus pleuropneumoniae*, *Mycoplasma hyopneumoniae*, *Pasteurella multocida* and *Streptococcus suis* when transferred to fattening units at 11 weeks of age, but displayed high levels of serum antibodies to *A. pleuropneumoniae* and to *P. multocida* 8 weeks later. Porcine circovirus type 2 (PCV2) was known to be present in both herds but there were no clinical signs of PCV2-associated diseases. All pigs were aged 9–11 weeks when included in the contact exposure experiment.

### In vitro exposure to Matrix-M

The transcriptional response to Matrix-M was studied in vitro using PBMCs collected from SPF pigs (Lövsta Research Station, Uppsala, Sweden). The PBMCs were isolated from heparinized blood by centrifugation for 45 min at 500*g* on FicollPaque PLUS (GE Healthcare Bio-Sciences, Uppsala, Sweden), washed twice in PBS and resuspended in RPMI 1640 medium supplemented with 20 mM HEPES buffer, 2 mM l-glutamine, 200 IU penicillin/mL, 100 μg streptomycin/mL, 50 μM 2–mercaptoethanol and 5% FCS (Gibco, Life Technologies, Carlsbad, CA, USA). Cell viability was determined with Trypan blue exclusion test and 10–20 × 10^6^ PBMCs were seeded into 6-well plates (Nunclon, Nunc, Roskilde, Denmark) for further enrichment into subpopulations.

Monocytes were enriched from PBMCs by plastic adherence for 2/3 h. Thereafter the non-adherent lymphocyte-enriched cells (referred to as “lymphocytes”) were transferred to new 6-well plates. Monocyte-derived dendritic cells (MoDCs) were generated as previously described [29] by culturing monocytes for 5 days in the presence of rpIL-4 (40 ng/mL; R&D Systems, Minneapolis, MN, USA) and rpGM-CSF (20 ng/mL; R&D Systems), replacing one-third of the culture volume after 3 days. The cells were used after 5 days when they had acquired a dendritic morphology. The resulting cell cultures were exposed to Matrix-M (1 μg/mL; Isconova AB, Uppsala, Sweden) that is delivered as a solution in PBS and easily blended into the culture medium. Lymphocytes were cultured for 3 days, with Matrix-M present for the last 6 h or 3 days. Monocytes were cultured for 1 day, with Matrix-M present for the last 6 h. MoDCs were exposed to Matrix-M for 6 h after generation, or with Matrix-M present during the 5 days of generation. All cultures were grown in 2 mL of medium for indicated durations at 37 °C with 7% CO_2_ in air.

### Matrix-M administration and contact exposure of SPF pigs

**Figure 1:**
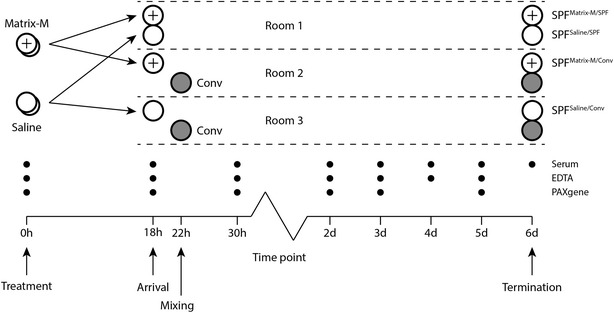
**Experimental design.** Sixteen SPF pigs were treated (0 h) with Matrix M (*n* = 8) or saline (*n* = 8) the evening before a 2-h transport to the animal facility. At arrival (18 h), the SPF pigs were allotted to three rooms. SPF pigs given Matrix-M or saline were mixed in the first room (SPF^Matrix-M/SPF^ and SPF^Saline/SPF^; *n* = 4 + 4). Four hours later (22 h) eight conventionally reared pigs (Conv) arrived and were mixed with four of the SPF pigs given Matrix-M (SPF^Matrix-M/Conv^) or four of those given saline (SPF^Saline/Conv^; *n* = 4) in the other two rooms. Blood samples were collected from the SPF pigs in tubes without additives (serum), EDTA tubes and PAXgene Blood RNA tubes as indicated. Clinical examination was performed concurrently with blood sampling and at least once more each day. Post-mortem examination was performed on all pigs at termination of the experiment (6 days).

All pigs in the contact exposure experiment were castrated males, aged 9–11 weeks. Two barrows from each of four SPF-litters received 150 μg Matrix-M (*n* = 8) suspended in 1 mL sterile endotoxin-free 0.9% NaCl solution (saline; Fresenius Kabi, Uppsala, Sweden), and two other barrows in these litters received saline only (*n* = 8). The intramuscular injection was made into the thigh at equal distance from the knee and the ischial tuberosity. After 16 h, all SPF pigs were transported during 2 h to the National Veterinary Institute. At arrival (18 h), the SPF pigs were allotted to four experimental groups and allocated to three rooms. Four hours later (22 h), four of the eight conventionally reared pigs from four litters were mixed with four pigs treated with Matrix-M (SPF^Matrix-M/Conv^) and with four pigs treated with saline (SPF^Saline/Conv^), respectively, in different rooms according to a split-litter design. The remaining eight SPF pigs (SPF^Matrix-M/SPF^ and SPF^Saline/SPF^) were housed in a third room. Thus, four experimental groups of four SPF pigs were established in three rooms with eight pigs in total per room (Figure 1). Each room of eight square metres, had individual ventilation and manure handling systems and an individual atrium for staff access. Pigs were fed 2% of their bodyweight with a commercial dry feed twice daily, and had free access to water and bedding material. The experiment was approved by the Uppsala Ethical Committee on Animal Experiments (Reg. No. C 105214/15).

The health status of the animals was recorded daily and graded as 0 = healthy; 1 = moderately affected; 2 = clearly affected; 3 = severely affected. Clinical signs of disease and respiratory signs were scored from 0 (none) to 3 (severe) as described [30]. Blood was collected by jugular venepuncture using vacuum tubes without additives or with EDTA (BD Vacutainer; BD Diagnostics, Franklin Lakes, NJ, USA), or PAXgene Blood RNA tubes (PreAnalytiX, Hombrechtikon, Switzerland), at time points indicated in Figure 1. Five days after mixing, all pigs were sacrificed by electrical stunning and subsequent exsanguination. Macroscopic alterations of the draining lymph node, bronchial lymph node, lung and joints were scored from 0 (none) to 3 (severe).

### RNA extraction

RNA from the in vitro exposure experiment was extracted as described [31] using a combination of Trizol reagent (Invitrogen, Carlsbad, CA, USA) and RNA purification spin columns (E.Z.N.A. Total RNA Kit, Omega Biotek, Norcross, GA, USA). RNA from blood collected in PAXgene tubes during the in vivo experiment was extracted using the PAXgene Blood RNA Kit (PreAnalytiX) and samples with low RNA yield were concentrated with the E.Z.N.A. MicroElute RNA Clean Up Kit (Omega Biotek). RNA concentration (A_260_) and purity (A_260/280_ and A_260/230_) was determined by spectrophotometry (NanoDrop 8000, NanoDrop Technologies, Montchamin, DE, USA). Integrity of RNA was determined by capillary gel electrophoresis (Experion, BioRad, Hercules, CA, USA).

### Synthesis of cDNA

Total RNA (250–1000 ng) from the in vitro and in vivo experiments were treated with RQ1 RNase-Free DNase (Promega, Madison, WI, USA) and DNA-free DNA Removal Kit (Life Technologies), respectively, followed by synthesis of cDNA with the GoScript Reverse Transcription System (Promega). Each cDNA was diluted five times in nuclease free water and stored at −20 °C until analysis.

### Real-time quantitative PCR (qPCR)

Transcripts specific for the genes CXCL8, IFITM3, IFNA, IFNB, IFNG, IL1B, IL6, IL10, IL12B, IL17A, SPP1, STING, TGFB1, TLR2, TLR4 and TNFA were quantified by qPCR using the QuantiTect SYBR Green PCR Kit (Qiagen) and an iQ5 Real Time PCR cycler (Bio-Rad). Each sample was amplified in duplicate 25 μL reactions with 2 μL cDNA (diluted × 5) and primers according to Table 1. The cycling conditions consisted of an initial cycle of 95 °C for 15 min followed by 40 cycles of 95 °C for 15 s, the gene-specific annealing temperature (Table 1) for 30 s and 72 °C for 30 s. A melt-curve analysis was done at the end of the program for product verification.

**Table 1:** **Primer details and qPCR conditions**

Gene	Primer sequence	Anneal temp (°C)	Primer conc (nM)	Eff (%)^a^	*r* ^2^	Melt point (°C)	References
CXCL8	F: AGCCAGGAAGAGACTAGAAAGAAA R: TTGGGGTGGAAAGGTGTG	56	500	97	0.998	81.5	New design
GAPDH^b^	F: ACACTCACTCTTCTACCTTTG R: CAAATTCATTGTCGTACCAG	56	500	94	0.998	80	[54]
HPRT^b^	F: GGTCAAGCAGCATAATCCAAAG R: CAAGGGCATAGCCTACCACAA	60	500	100	0.996	80	[55]
IFITM3	F: ATCAACATCCGAAGCGAGACC R: GGAAAATTACCAGGGAGCCAGTG	56	500	96	0.999	85.5	New design
IFN-α	F: AGCCTCCTGCACCAGTTCTG R: TCACAGCCAGGATGGAGTCC	60	500	100	0.997	84.5	[20]
IFN-β	F: TAGCACTGGCTGGAATGAAACC R: TCAGGTGAAGAATGGTCATGTCT	58	400	104	0.993	79.5	[20]
IFN-γ	F: TGGTAGCTCTGGGAAACTGAATG R: GGCTTTGCGCTGGATCTG	60	400	102	0.998	76.5	[31]
IL1B	F: GTGATGGCTAACTACGGTGACAA R: CTCCCATTTCTCAGAGAACCAAG	60	400	91	0.999	79.5	[56]
IL6	F: CTGGCAGAAAACAACCTGAACC R: TGATTCTCATCAAGCAGGTCTCC	60	400	98	0.994	77.5	[57]
IL10	F: CGGCGCTGTCATCAATTTCTG R: CCCCTCTCTTGGAGCTTGCTA	60	400	100	0.995	80.5	[57]
IL12B	F: TCTTGGGAGGGTCTGGTTTG R: AAGCTGTTCACAAGCTCAAGTATGA	61	400	96	0.999	76.5	[31]
IL17A	F: CAGACGGCCCTCAGATTACTCCA R: AGCCCACTGTCACCATCACTTTCT	61	400	91	0.993	84.5	New design
PPIA^b^	F: GCAGACAAAGTTCCAAAGACAG R: AGATGCCAGGACCCGTATG	60	400	92	0.999	80.5	[58]
RPL32^b^	F: CGGAAGTTTCTGGTACACAATGTAA R: TGGAAGAGACGTTGTGAGCAA	55	300	97	0.997	77	[20]
SPP1	F: TTGGACAGCCAAGAGAAGGACAGT R: GCTCATTGCTCCCATCATAGGTCTTG	56	300	93	0.997	82.5	[20]
STING	F: TTACATCGGGTACCTGCGGC R: CCGAGTACGTTCTTGTGGCG	56	500	101	0.992	82	[20]
TGFB1	F: TACGCCAAGGAGGTCACCC R: CAGCTCTGCCCGAGAGAGC	60	400	90	0.992	83	[59]
TLR2	F: GGCAAGTGGATTATTGACAACATC R: ACCACTCGCTCTTCACAAAGTTC	60	500	94	0.997	78.5	[60]
TLR4	F: CTTCACTACAGAGACTTCATTC R: ACACCACGACAATAACCT	54	500	89	0.999	79	[61]
TNFA	F: AGCCTCTTCTCCTTCCTCCTG R: GAGACGATGATCTGAGTCCTTGG	60	400	91	0.993	84	[31]
YWHAZ^b^	F: ATTGGGTCTGGCCCTTAACT R: GCGTGCTGTCTTTGTATGACTC	58	400	101	0.997	78.5	[20]

Conditions optimized or re-optimized for reagents and equipment described in “Materials and methods”.

^a^PCR efficiency estimated on serial dilutions of reference cDNA.

^b^Reference gene used for normalization.

To evaluate the level of genomic DNA contamination in the samples, the IFNA assay was used either on aliquots run in the cDNA synthesis without reverse transcriptase (in vitro samples) or on the DNased total RNA at a concentration taking into account the dilution factor generated at cDNA synthesis (in vivo samples).

For initial analyses, cDNA from four pigs each in two experimental groups (SPF^Matrix-M^ and SPF^Saline^) was pooled within each group and screened using a custom made (Additional file 1) dried-down qPCR plate array (PrimePCR, Bio-Rad). The expression of selected genes from the plate were further analysed in wet format (IL18, MYD88, NLRP3 and TLR9). All PrimePCR assays were run on the CFX96 Touch (Bio-Rad) using SsoAdvanced Universal SYBR^®^ Green Supermix (Bio-Rad) according to the manufacturer’s instructions. All reactions on the custom made plate were run as single reactions whereas assays in wet format were run in duplicates.

### Reference genes and calculation of relative expression

Data acquired from the in vitro experiment was normalized to the reference genes RPL32 and YWHAZ [20]. For the in vivo experiment, a total of five reference genes were evaluated (GAPDH, HPRT, PPIA, RPL32, YWHAZ), of which GAPDH, RPL32 and YWHAZ were present on the qPCR plate array. Based on geNorm analysis within the program qBasePLUS (Biogazelle), PPIA and RPL32 were selected for normalization of data (*M* = 0.345). Relative gene expression (fold change, FC) in samples was calculated to a calibrator, either untreated controls (in vitro) or the 0 h time point (in vivo), and normalized [32] to the aforementioned reference genes

### DNA extraction and screening for PCV2

Blood samples from SPF pigs (0, 18 h or 5 days after mixing) and conventionally reared pigs (22 h or 5 days after mixing) were screened for presence of PCV2. DNA from 200 µL blood collected with EDTA as additive was extracted using the QIAamp DNA mini kit (Qiagen, Hilden, Germany), eluted in 100 µL elution buffer and the concentration and purity was determined by spectrophotometry (Nanodrop 8000). The undiluted DNA was screened using a universal PCV2 assay (F: TAATTTTGCAGACCCGGAAAC; R: AGTAGATCATCCCACGGCAG) against the ORF1 reading frame [33] and the QuantiTect SYBR Green PCR Kit (Qiagen) as described above (56 °C annealing temperature; 96% efficiency). The presence of PCR inhibitors was evaluated by spiking selected negative samples with 2 × 10^5^ copies of PCV2 DNA before analysis.

### Blood and serum analyses

Total and differential white blood cell (WBC) counts were determined in blood collected with EDTA as additive using an automated cell counter (Exigo Veterinary Hematology System; Boule Diagnostics, Spånga, Sweden). Blood samples without additives were centrifuged for 10 min at 800 *g* and serum was collected and stored at −20 °C until analysis. Serum amyloid A was determined using a commercial kit (Phase range Serum Amyloid A Assay; Tridelta Development, Maynooth, Ireland) according to the manufacturer’s instructions. Serum antibodies to *M. hyopneumoniae* and *H. parasuis* were analysed by commercial kits according to the manufacturers’ instructions (IDEXX *M. hyo*. Ab test, IDEXX, Westbrook, USA; Swinecheck HPS, Biovet, St Hyacinthe, Canada) and to *A. pleuropneumoniae* and *P. multocida* by indirect ELISA systems [30, 34].

### Data and statistical analysis

Statistical analyses were performed in Excel (Microsoft) or Prism 5.0 (GraphPad Software). WBC counts are shown as mean ± SD and statistical differences analysed by Student’s t test. Gene expression is reported as fold change (FC) and shown as median or geometrical mean with SD. Statistical differences in gene expression between experimental groups or treatments were determined using the Mann–Whitney U test or, where applicable, the Wilcoxon matched-pairs signed rank test. Spearman correlation was calculated between gene expression and WBC counts. For all tests, *p* values less than 0.05 were regarded as significant and a gene with a FC < 0.5 or > 2 was considered as differentially expressed.

## Results

### Transcriptional response to Matrix-M in vitro

The transcriptional response in lymphocytes, monocytes and MoDCs was measured after short-term (6 h) and long-term (3 or 5 days) stimulation with Matrix-M (Table 2). During these conditions, gene expression of the pro-inflammatory cytokines IL-1β and CXCL8, but not TNF-α or IL-6, was up-regulated by Matrix-M in both lymphocytes and monocytes. Increased transcription of IL-6 was only detected in MoDCs that had been generated for 5 days in the presence of Matrix-M.

**Table 2:** **Effects of Matrix-M on gene expression (relative gene expression versus untreated control) in cultures of porcine monocytes, lymphocytes and monocyte-derived dendritic cells (MoDC)**

Target name	Lymphocytes 3 days^a^		Monocytes 1 day^a^	MoDC 5 days^a^	
	Matrix-M 6 h^b^	Matrix-M 3 days^b^	Matrix-M 6 h^b^	Matrix-M 6 h^b^	Matrix-M 5 days^b^
	*n* = 8^c^	*n* = 8^c^	*n* = 10^c^	*n* = 6^c^	*n* = 6^c^
IL-1β	2.0	9.6*	3.3*	–	–
IL-6	< >	3.9	2.1	< >	10.2*
TNF-α	2.5	< >	< >	–	–
CXCL8	2.4*	5.9**	3.2**	< >	< >
IL-12p40	2.6*	2.6*	nd	–	–
IL-17A	0.3	27.8*	nd	–	–
IFN-γ	< >	4.8*	nd	–	–
IL-10	0.2*	0.4*	< >	–	–
TGF-β	< >	< >	–	–	–
IFN-α	< >	< >	< >	< >	3.8*
IFN-β	nd	nd	nd	< >	2.8
IFITM3	< >^d^	< >^d^	< >	< >^d^	< >^d^
SPP1	< >^d^	< >^d^	< >^e^	< >^d^	0.2^d^
STING	< >^d^	< >^d^	–	< >^d^	< >^d^
TLR2	0.4**	0.2	< >	0.3*	0.2*

< >, gene not affected, i.e. 0.5 < FC < 2.

*nd* not detected; too few samples showed any expression of the gene of interest to allow FC calculation, – not analysed.

* *p* < 0.05 and ** *p* < 0.01 for difference in expression between Matrix-M treated samples and medium control (i.e. FC ≠ 1); Wilcoxon matched-pairs signed rank test.

^a^Total culture time.

^b^Matrix-M exposure time.

^c^Unless otherwise indicated.

^d^ *n* = 4.

^e^ *n* = 6.

Expression of the T_H_-related cytokine genes IL-12p40, IL-17A and IFN-γ was increased in lymphocytes cultured for 3 days in the presence of Matrix-M. These long-term exposed lymphocytes displayed a decreased transcription of IL-10. IFN-α transcription was up-regulated in MoDCs but not in any other cell type. IFN-β, the interferon-regulated gene IFITM3 and the type I interferon-associated genes STING and SPP1 remained unaffected by Matrix-M. The expression of TLR2 was down-regulated in lymphocytes and in MoDCs exposed to Matrix-M.

In summary, in vitro exposure to Matrix-M affected the gene expression for immunomodulatory cytokines, including IFN-α in cultures of porcine blood mononuclear cells. These effects were most pronounced in long-term cultures, but were also indicated in cultures exposed to Matrix-M for 6 h.

### Transcriptional response to Matrix-M in SPF pigs

**Figure 2:**
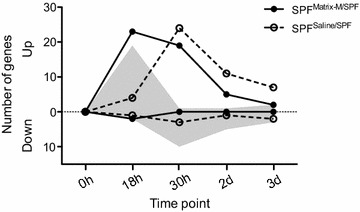
**Gene regulation in pooled blood samples from pigs administered Matrix-M or saline.** The number of up-regulated and down-regulated genes (fold change > 2 and < 0.5, respectively) in pooled blood from pigs administered Matrix-M (*n* = 4) or saline (*n* = 4), as analysed using a 92-gene qPCR plate array. The expression analysis is presented as the number of differentially expressed genes at each time point in relation to the 0 h time point (Matrix-M solid circles, saline open circles) or as the number of differentially expressed genes in Matrix-M pigs (up-regulated or down-regulated) in relation to the expression in saline pigs at each corresponding time point (depicted by the grey area).

The transcriptional response to Matrix-M was followed during 5 days in blood obtained from SPF pigs that were administered Matrix-M (SPF^Matrix-M/SPF^; *n* = 4) or saline (SPF^Saline/SPF^; *n* = 4) and subjected to a 2-h transport. Pooled samples from pigs administered Matrix-M (*n* = 4) were compared to pooled samples from pigs given saline (*n* = 4) using a qPCR plate array containing 92 genes representing innate immunity parameters. A transient increase in gene expression (FC > 2 compared to 0 h) was found for both groups after transport (Figure 2; Additional file 1). The increase appeared earlier in pigs administered Matrix-M (23 genes at 18 h, 19 genes at 30 h) than in those given saline (4 genes at 18 h, 24 genes at 30 h). Only a couple of genes were down-regulated (FC < 0.5) over this time period; CXCL10 and TLR7 in the Matrix-M group and TNFA and FOXP3 in the saline group. Comparing FC values between groups at 18 h confirmed a higher expression (> twofold) of 19 genes in the Matrix-M treated pigs (C3, CCR1, CD80, FOXP3, IFNGR1, IL18, IL1A, IRF7, JAK2, MYD88, NFKBIA, NLRP3, NOD1, NRAMP1, STAT3, TLR4, TLR8, TLR9 and TYK2).

**Figure 3:**
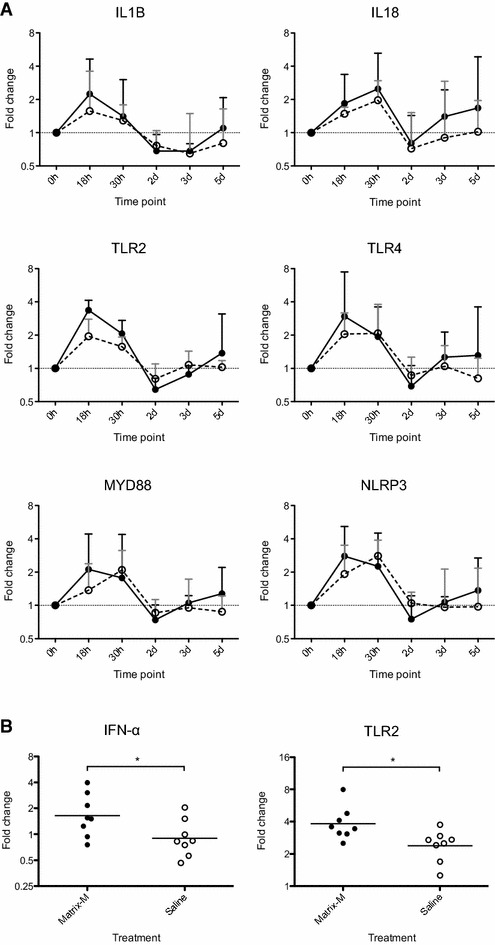
**Gene expression in blood of SPF pigs administered Matrix-M (solid line) or saline (open line). A** Alterations of the gene expression over a 5 day period following administration of Matrix-M or saline (0 h), transport and mixing with non-littermates (18 h). FC values at indicated times are calculated against the 0 h time point and given as geometric mean ± SD, *n* = 4. **B** Gene expression for all SPF pigs, measured 18 h after injection of Matrix-M (*n* = 8) or saline (*n* = 8). FC values are calculated against the 0 h time point and individual FC values and geometric mean are given for each treatment.

Based on results from the pooled samples and the in vitro data, IFN-α, IFN-β, CXCL8, IL1B, IL6, IL18, MYD88, NLRP3, TLR2, TLR4 and TLR9 were selected for analysis of gene expression in individual samples. The gene expression of MYD88 and NLRP3 displayed a similar kinetic as depicted by the qPCR plate array, i.e., an earlier increase in pigs administered Matrix-M compared to those given saline (Figure 3A). In contrast, the expression of IL1B, TLR2 and TLR4 peaked at 18 h in both groups, whereas the expression of IL18 was highest at 30 h. Transcripts specific for CXCL8, IFN-β and IL-6 were hardly detected and the average FC values for IFN-α and TLR9 did not exceed 2 in blood from the SPF pigs, regardless of treatment. However, statistical analysis of the gene expression data including samples from all SPF pigs at 18 h, i.e. before contact exposure, revealed a significantly (*p* < 0.05) higher expression of IFN-α and TLR2 in pigs administered Matrix-M than in those given saline (Figure 3B). At autopsy, the draining lymph node was enlarged in four of the eight pigs injected with Matrix-M, indicating cell recruitment and/or cell proliferation (Table 3). No such reactions were recorded for any pig injected with saline.

**Table 3:** **Clinical score and post-mortem findings recorded for the SPF pigs in the natural challenge experiment**

Pig ID	Clinical findings					Post-mortem examination				
	General condition		Respiratory signs		Other	DLN	Lung	BLN	Joints	Other
	Score^a^	Onset^b^ (day)	Score^a^	Onset^b^ (day)						
SPF^Matrix-M/SPF^										
#1	–	–	–	–	Lameness^c^	–	–	–	–	Abscess^d^
#2	–	–	–	–	–	–	–	–	–	–
#3	–	–	–	–	–	2	–	–	–	–
#4	–	–	–	–	–	–	–	–	–	–
SPF^Saline/SPF^										
#5	–	–	–	–	–	–	–	–	–	–
#6	–	–	–	–	–	–	–	–	–	–
#7	–	–	–	–	–	–	–	–	–	–
#8	–	–	–	–	–	–	–	–	–	–
SPF^Matrix-M/Conv^										
#9	–	–	0.5	1	–	–	0.5	1	–	–
#10	–	–	0.5	1	–	1	–	1	–	–
#11	–	–	0.5	2	–	1	0.5	–	–	–
#12	–	–	1	3	–	1	0.5	–	–	–
SPF^Saline/Conv^										
#13	–	–	0.5	1	–	–	0.5	0.5	–	–
#14	2	3	1	1	Lameness^e^	–	3	1	3	Ascites^f^
#15	–	–	1	2	–	–	–	1	–	–
#16	1	2	1	1	Lameness^c^	–	–	–	2	–

Pigs were administered Matrix-M (Nos. 1–4 and 9–12) or saline (Nos. 5–8 and 13–16) and were challenged by contact exposure (Nos. 9–16) or mixed with non-littermate SPF pigs (Nos. 1–8).

*DLN* draining lymph node, *BLN* bronchial lymph node.

^a^Maximum score recorded.

^b^Days after mixing.

^c^Onset 4 days after mixing.

^d^Superficial abscess of 1 cm at fetlock joint.

^e^Onset 3 days after mixing.

^f^Ascetic fluid in the peritoneum.

### Clinical effects of Matrix-M in SPF pigs at contact exposure

To evaluate if the indicated immune activation by Matrix-M had any clinical effect, SPF pigs where challenged by contact exposure to conventionally reared pigs 22 h after administration of Matrix-M (SPF^Matrix-M/Conv^, Nos. 9–12) or saline (SPF^Saline/Conv^, Nos. 13–16). The contact pigs were clinically healthy during the trial but lung lesions were recorded post-mortem in five of them (two out of four mixed with SPF^Matrix-M/Conv^ and three out of four mixed with SPF^Saline/Conv^ pigs). All conventionally reared pigs were seronegative to *H. parasuis*, whereas antibodies were detected in sera collected from eight SPF pigs originating from two litters. These antibody levels decreased significantly from day 0 to day 6 (*p* < 0.05; paired *t* test), indicating that they were of maternal origin. Both contact pigs and SPF pigs remained seronegative to *M. hyopneumoniae*, *A. pleuropneumoniae* and *P. multocida* throughout the study. The experimental conditions did not affect the PCV2 viral load as no PCV2 DNA was detected in blood from any of the SPF or contact pigs before or at the end of the experiment.

No signs of respiratory disease or lesions in the respiratory tract were recorded for SPF pigs that were mixed with other SPF pigs (SPF^Matrix-M/SPF^ and SPF^Saline/SPF^). None of the SPF pigs that were mixed with conventional pigs at 22 h showed any clinical signs of disease before the contact exposure. Thereafter all these SPF-pigs, irrespective of treatment, developed respiratory symptoms within 1–3 days that lasted throughout the study (Table 3). At necropsy, seven out of these eight SPF pigs displayed reactions in bronchial lymph nodes and/or lung. Lameness and decreased general condition were recorded in two out of the four SPF pigs administered saline (Nos. 14 and 16), with onset 2–4 days after mixing. These two pigs were the only ones displaying joint lesions at the post-mortem examination. In addition, ascetic fluid was found in the peritoneum of pig no. 14.

Thus, all challenged SPF pigs developed respiratory disease, but affected general health status and joint lesions were only recorded for pigs administered saline before the contact exposure. The draining lymph node was enlarged in four of the eight pigs receiving Matrix-M (Nos. 3, 10, 11, 12) but not in any pig administered saline. One SPF^Matrix-M/SPF^ pig (No. 1) turned lame on day 5, which was associated to a superficial abscess in the fetlock joint.

### Granulocyte counts, SAA levels and gene expression in Matrix-M treated SPF pigs during the contact exposure experiment

**Figure 4:**
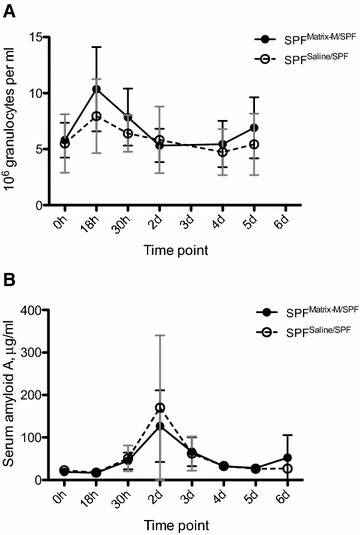
**Granulocyte counts and SAA levels in blood of SPF pigs administered Matrix-M or saline. A** Alterations in granulocyte counts over a 5 day period following administration of Matrix-M or saline (0 h), transport and mixing with non-littermates at 18 h. Mean value ± SD, n = 4. **B** Alterations in SAA over a 5 day period following administration of Matrix-M or saline (0 h), transport and mixing with non-littermates at 18 h. Mean value ± SD, *n* = 4.

Granulocyte counts, SAA levels and gene expression were monitored for all SPF-pigs throughout the study. The granulocyte numbers increased after transport (18 h) in all SPF groups, from 5.5 ± 1.4 × 10^6^ to 10.6 ± 3.3 × 10^6^/mL blood in pigs treated with Matrix-M (*n* = 8) and from 5.6 ± 2.2 × 10^6^ to 8.9 ± 3.3 × 10^6^/mL blood in pigs given saline (*n* = 8). The granulocyte numbers then returned to baseline and remained so in SPF pigs mixed with other SPF pigs (Figure 4A), except in the pig with an abscess in the fetlock joint (pig no. 1), that had 10.5 × 10^6^ granulocytes/mL blood at day 5. Increased levels of SAA were observed in all SPF groups at day 2, i.e. 1 day after allocation and mixing with other pigs. The SAA levels returned to baseline by day 4 in SPF pigs that were mixed with other SPF pigs (Figure 4B), but increased to 132 μg/mL in blood collected on day 6 from pig no. 1. This pig displayed increased gene expression of MyD88, IL18, NLRP3, TLR2 and TLR4 in blood collected day 5. No such alteration in gene expression was recorded for any of the other SPF pigs mixed with SPF-pigs, irrespective of treatment.

**Figure 5:**
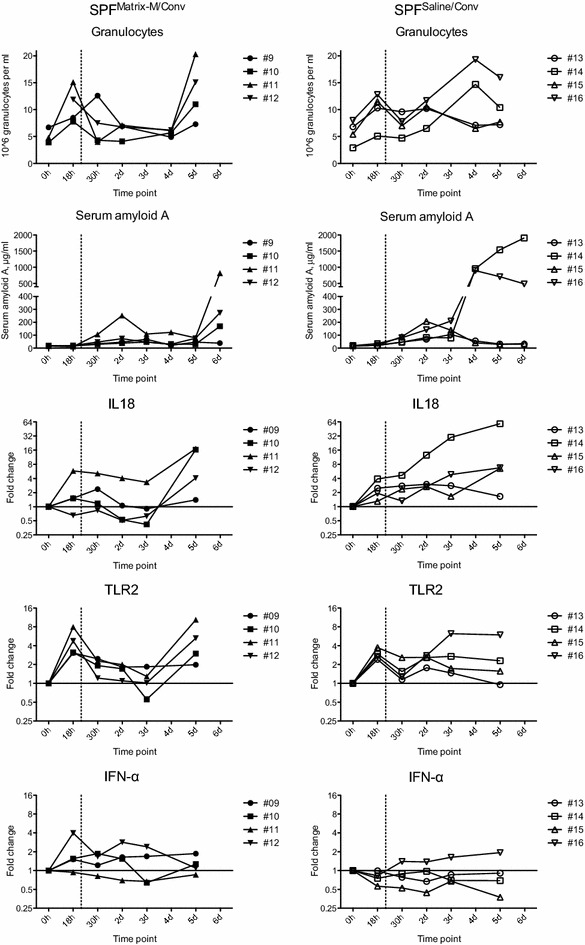
**Granulocyte counts, SAA and gene expression in blood of SPF pigs administered Matrix-M or saline.** The pigs were administered Matrix-M or saline (0 h), transport to the animal facility (arrival at 18 h) and exposed to conventional pigs (at 22 h, dashed vertical line). Individual FC values are presented for SPF^Matrix/Conv^ pigs (closed symbols) and SPF^Saline/Conv^ pigs (open symbols). FC is calculated against the 0 h time point for each individual.

A second increase in granulocyte number was recorded following the contact exposure of SPF pigs to conventionally reared pigs (Figure 5). Among SPF pigs administered saline (SPF^Saline/Conv^), the granulocyte counts remained elevated throughout the study and increased considerably for pig nos. 14 and 16 at days 4 and 5. In these two pigs the SAA levels increased above 500 μg/mL from day 4 and onwards. An increased expression of IL18 and TLR2 was also obvious in blood samples collected from these pigs at the last occasions of sampling, i.e. when they displayed lameness and depressed general condition.

The granulocyte numbers in blood from pigs administered Matrix-M (SPF^Matrix-M/Conv^) before the contact exposure remained at baseline until day 5 when it increased in three of the four pigs (Nos. 10, 11, 12). This increase coincided with an increase in both SAA levels and expression of IL18 and TLR2 (Figure 5) and IL1B, TLR4 and MYD88 (data not shown) in blood from these pigs. Pig no. 11 had the highest granulocyte count and SAA level on the last day recorded. Furthermore, it also had a higher expression of IL18 than the other pigs already at 18 h, which also was evident for MYD88 and NLRP3 (data not shown). In contrast, pig no. 9 had a modest increase in granulocyte numbers and no increase in SAA levels on day 5 and 6, and generally showed the lowest variation in gene expression.

The expression of the IFN-α gene was not affected by contact exposure in either SPF^Matrix-M/Conv^ pigs or SPF^Saline/Conv^ pigs during the study (Figure 5). Similarly, IFN-β and IL-6 transcripts were only detected in a few of the SPF pigs, which were mixed with other SPF pigs (data not shown). The expression for IL1B, TLR4, MYD88 and NLRP3 generally increased in all pigs at 18 h but thereafter the individual variations were too large for conclusive results (data not shown).

Taken together, the expression pattern of several of the pro-inflammatory genes analysed correlated with changes in granulocyte counts and SAA levels. This relationship was further corroborated by a significant correlation between granulocyte counts and the expression of *TLR2* (*p* < 0.001; rho = 0.63; Spearman analysis for all pigs and time points).

## Discussion

Immunomodulatory effects of Matrix-M were evaluated at contact exposure of SPF pigs to conventionally reared pigs. Although all contact SPF pigs in this model developed respiratory disease, only control pigs administered saline developed symptoms of systemic disease as evidenced by joint lesions. Granulocyte counts, levels of the acute-phase protein SAA and gene expression of IL-18 and TLR2 indicated a difference in kinetics of disease development between the two experimental groups. Pigs receiving intramuscular injection of Matrix-M, but not yet subjected to contact exposure, showed a statistically significant increase in expression of TLR2 and IFN-α in blood after 18 h.

Prior to the in vivo studies, in vitro effects of Matrix-M on partially enriched porcine mononuclear cell populations were evaluated. Clearly upregulated genes included proinflammatory cytokines (IL-1β, CXCL8), T_H_ cytokines (IFN-γ, IL-12p40, IL-17A) and IFN-α. This up-regulation was most pronounced in PBMCs devoid of adherent cells, i.e., lymphocytes, when cultured for 3 days in the presence of Matrix-M. Lymphocytes cultured for only 16 h and exposed to Matrix-M for 6 h did not change their expression of any of the genes analysed (data not shown), in agreement with previous results [20]. The lymphocyte preparations in the current study were only partially purified, with no attempts to phenotypic characterization of the cells. However, γδ T cells that can produce both IL-17 and IFN-γ reach high numbers in the porcine circulation [35, 36] that may have contributed to the relative increase in IL-17 and IFN-γ transcription in lymphocytes. IL-12 and IL-18 act synergistically on the IFN-γ production by porcine T cells [35] and IL-12p40 transcripts were detected in lymphocytes cultured in the presence of Matrix-M. Thus, both the period of exposure to Matrix-M and the total culture time as well as the cell composition appears important for the induction of cytokine gene expression by Matrix-M in vitro.

Most adjuvant effects are evaluated by in vivo or in vitro antigen re-stimulation. In such cases it is comparatively easy to profile the specific immunological response based on cytokine production, effector cell development and antibody isotypes. In the absence of antigen, adjuvant effects are solely monitored by innate immune parameters. However, innate immune reactions are influenced by mediators from several organs making them more problematic to assess in vitro. Using defined immunomodulators whole blood cultures have been used with some success to monitor cytokine profiles in man [37] and inflammatory biomarkers in pigs [38]. Still, the in vitro system does not always reflect the in vivo situation even in the case when defined immunomodulating molecules are used. PolyI:C and plasmid DNA both readily induced IFN-α in porcine tissue whereas these substances only induced low levels of IFN-α in porcine PBMC cultures, unless the inducers were pre-incubated with lipofectin [39, 40]. In accordance, previous in vivo characterization of the immunomodulatory capacity of ISCOM-Matrix formulations, such as production of cytokines and activation of dendritic cells, has not been successfully reproduced in vitro [41].

The transcriptional response to Matrix-M was further evaluated in vivo. For that purpose SPF pigs were administered Matrix-M or saline 16 h before a 2-h transport and subsequent mixing with contact pigs. In line with earlier observations [42, 43], the transport and mixing caused a transient increase in granulocyte counts (at 18 h) and SAA levels (at day 2) with no differences between pigs administered Matrix-M and those given saline. These effects were therefore attributed to the “stress” all pigs experienced. An increased transcription appeared for approximately 20% of the 92 innate immunity genes assessed in the pooled blood samples. This increase was evident in pooled samples collected both at 18 and 30 h from SPF^Matrix-M^ pigs but only found at 30 h in pooled samples from SPF^Saline^ pigs. Of these genes only two (NFKBIA and S100A8) were identified as differentially expressed in a whole transcriptomic analysis of blood cells from pigs after ACTH administration [44]. However, SAA, IL-6, IgA and IL-18 are all suggested as biomarkers of “stress” in the pig [45]. Furthermore, studies in man indicate an intrinsic network between glucocorticosteroids, SAA, cytokines, chemokines, TLR2 and TLR4 [46]. Thus, it is likely that the physiological effect of transport masked effects on the transcription of innate immune genes caused by Matrix-M administration.

When individual samples at time point 18 h were analysed, only the expression of IFN-α and TLR2 was significantly increased in pigs administered Matrix-M compared to saline. This increased gene expression of IFN-α is in line with the induction of interferon-regulated genes in muscle and draining lymph node from pigs 24 h after administration of Matrix-M [16]. Subsequent analyses also demonstrated IFN-β transcripts as well as a low expression of SPP1 and STING in some of these lymph nodes [20]. When analysed in vitro, IFNB, STING and SPP1 showed no expression or were down-regulated whereas IFN-α was up-regulated in long-term cultures of MoDC. Similarly, the expression of TLR2 was down-regulated when analysed in vitro but significantly up-regulated in blood from the SPF-pigs administered Matrix-M. TLR2 was also highly up-regulated locally after intramuscular injection with Matrix-M in pigs [16] and analysis on archived material from that study revealed a tendency toward up-regulation of TLR2 in PBMCs isolated 17 h after the injection (data not shown). In blood, porcine TLR2 is mostly expressed on monocytes, with some expression on granulocytes and no expression on lymphocytes [47]. Thus, changes in the blood relative cellular composition for example caused by altered mononuclear cell migration and polymorphonuclear cell influxes need to be considered amongst other factors when evaluating gene expression in blood samples [48]. Differences between adjuvant effects recorded in vitro and in vivo may hinder the evaluation of mechanistic effects [49] and co-culture systems with endothelial cells can better reflect the in vivo situation when using PBMC for evaluation of immune activators [50].

Based on the results from in vitro data and previous reports on the immunostimulatory effect of Matrix-M [16] we hypothesized that Matrix-M could act prophylactic or therapeutic to affect the clinical outcome of a natural infection. Experience from porcine reproductive and respiratory syndrome virus [22] and PCV2 [51] demonstrate that infectious diseases in production animals are often multifactorial and may benefit from stressors such as weaning, transport and mixing of groups [21]. In an effort to imitate field conditions, SPF pigs were subjected to non-infectious stress from transport and mixing of groups and to infectious stress by contact exposure to conventionally reared pigs (Figure 1). Two of the SPF pigs that were administered saline before contact exposure developed signs of systemic disease that resembled Glässer’s disease [52]. This syndrome is caused by *H. parasuis* infection and is commonly associated with transport and mixing of pigs of different health status. Post-mortem examination supported this diagnosis, but *H*. *parasuis* could not be recovered from affected organs by cultivation. However, the presence of *H. parasuis* was demonstrated by metagenomic sequencing of 16S rRNA genes (SciLifeLab, Uppsala, Sweden, data not shown) and the low level of serum antibodies to *H. parasuis* indicated that the pigs were at risk to develop disease. The affected pigs expressed high levels of SAA and granulocyte counts following the onset of clinical symptoms. Notably, the SAA levels correlated with severity of disease, as outlined in [53], as well as to the expression of IL18 and TLR2. The SAA levels and granulocyte counts returned to baseline after the initial peak in all other SPF pigs mixed with conventional pigs, irrespective of treatment. Thus, there appeared no systemic inflammation in these pigs, although all SPF pigs developed respiratory symptoms. However, in three out of four challenged SPF pigs receiving Matrix-M, both SAA levels, granulocytes counts and transcription of IL1B, IL18, MYD88, NLRP3, TLR2 and TLR4 increased on the last day recorded. All these parameters were also increased in the control pig (No. 1) suffering from an abscess, further supporting their use as indicators of inflammation. This suggests that Matrix-M did not fully protect against systemic disease, but affected the disease kinetics as innate immune responses appeared later after challenge in Matrix-M injected pigs (Figure 5).

Taken together, both the in vitro and in vivo results indicate an immunomodulatory effect of Matrix-M in the pig. In the contact exposure model, Matrix-M seemed to be able to reduce or delay systemic symptoms resembling Glässer’s disease. There was no discernible effect on local respiratory symptoms but this might be overcome by employing a different route of administration. If these indications can be confirmed in the field, Matrix-M could be suitable to enhance innate immune responses during critical moments in pig management. Equally important, the capacity to a rapid activation of innate immune parameters is highly desirable in adjuvants used for emergency vaccination.

## Additional file

**Additional file 1.** **Gene expression in pooled blood samples from pigs administered Matrix-M or saline.** The gene expression was analysed by a qPCR plate array in pooled blood samples collected at 0, 18, 30 h, 2 and 3 days after administration of Matrix-M (4 pigs) or saline (4 pigs). The relative gene expression is given as FC for each experimental group relative to 0 h (FC = 1). These values are then used to compare the gene expression after Matrix-M administration in relation to that after administration of saline.

## References

[1] de Paula Barbosa A (2014). Saponins as immunoadjuvant agent: a review. Afr J Pharm Pharmacol.

[2] McKee AS, MacLeod MK, Kappler JW, Marrack P (2010). Immune mechanisms of protection: can adjuvants rise to the challenge?. BMC Biol.

[3] Sun HX, Xie Y, Ye YP (2009). Advances in saponin-based adjuvants. Vaccine.

[4] Rajput ZI, Hu SH, Xiao CW, Arijo AG (2007). Adjuvant effects of saponins on animal immune responses. J Zhejiang Univ Sci B.

[5] Morein B, Hu KF, Abusugra I (2004). Current status and potential application of ISCOMs in veterinary medicine. Adv Drug Deliv Rev.

[6] Lövgren K, Morein B (1988). The requirement of lipids for the formation of immunostimulating complexes (ISCOMS). Biotechnol Appl Biochem.

[7] Pedersen GK, Sjursen H, Nøstbakken JK, Jul-Larsen Å, Hoschler K, Cox RJ (2014). Matrix M(TM) adjuvanted virosomal H5N1 vaccine induces balanced Th1/Th2 CD4(+) T cell responses in man. Hum Vaccines Immunother.

[8] Quinn KM, Yamamoto A, Costa A, Darrah PA, Lindsay RW, Hegde ST (2013). Coadministration of polyinosinic: polycytidylic acid and immunostimulatory complexes modifies antigen processing in dendritic cell subsets and enhances HIV gag-specific T cell immunity. J Immunol.

[9] Madhun AS, Haaheim LR, Nilsen MV, Cox RJ (2009). Intramuscular Matrix-M-adjuvanted virosomal H5N1 vaccine induces high frequencies of multifunctional Th1 CD4+ cells and strong antibody responses in mice. Vaccine.

[10] Bengtsson KL, Song H, Stertman L, Liu Y, Flyer DC, Massare MJ (2016). Matrix-M adjuvant enhances antibody, cellular and protective immune responses of a Zaire Ebola/Makona virus glycoprotein (GP) nanoparticle vaccine in mice. Vaccine.

[11] Tsuda T, Sugimura T, Murakami Y (1991). Evaluation of glycoprotein gII ISCOMs subunit vaccine for pseudorabies in pig. Vaccine.

[12] González AM, Nguyen TV, Azevedo MS, Jeong K, Agarib F, Iosef C (2004). Antibody responses to human rotavirus (HRV) in gnotobiotic pigs following a new prime/boost vaccine strategy using oral attenuated HRV priming and intranasal VP2/6 rotavirus-like particle (VLP) boosting with ISCOM. Clin Exp Immunol.

[13] Iosef C, Van Nguyen T, Jeong K, Bengtsson KL, Morein B, Kim Y (2002). Systemic and intestinal antibody secreting cell responses and protection in gnotobiotic pigs immunized orally with attenuated Wa human rotavirus and Wa 2/6-rotavirus-like-particles associated with immunostimulating complexes. Vaccine.

[14] Garcia JL, Gennari SM, Navarro IT, Machado RZ, Sinhorini IL, Freire RL (2005). Partial protection against tissue cysts formation in pigs vaccinated with crude rhoptry proteins of *Toxoplasma gondii*. Vet Parasitol.

[15] Xiong Q, Wei Y, Feng Z, Gan Y, Liu Z, Liu M (2014). Protective efficacy of a live attenuated *Mycoplasma hyopneumoniae* vaccine with an ISCOM-matrix adjuvant in pigs. Vet J.

[16] Ahlberg V, Bengtsson KL, Wallgren P, Fossum C (2012). Global transcriptional response to ISCOM-Matrix adjuvant at the site of administration and in the draining lymph node early after intramuscular injection in pigs. Dev Comp Immunol.

[17] Magnusson SE, Reimer JM, Karlsson KH, Lilja L, Bengtsson KL, Stertman L (2012). Immune enhancing properties of the novel Matrix-M adjuvant leads to potentiated immune responses to an influenza vaccine in mice. Vaccine.

[18] Reimer JM, Karlsson KH, Bengtsson KL, Magnusson SE, Fuentes A, Stertman L (2012). Matrix-M adjuvant induces local recruitment, activation and maturation of central immune cells in absence of antigen. PLoS One.

[19] Lund H, Boysen P, Åkesson CP, Lewandowska-Sabat AM, Storset AK (2016). Transient migration of large numbers of CD14(++) CD16(+) monocytes to the draining lymph node after onset of inflammation. Front Immunol.

[20] Fossum C, Hjertner B, Ahlberg V, Charerntantanakul W, McIntosh K, Fuxler L (2014). Early inflammatory response to the saponin adjuvant Matrix-M in the pig. Vet Immunol Immunopathol.

[21] Amadori M, Zanotti C (2016). Immunoprophylaxis in intensive farming systems: the way forward. Vet Immunol Immunopathol.

[22] Amadori M, Razzuoli E (2014). Immune control of PRRS: lessons to be learned and possible ways forward. Front Vet Sci.

[23] Dec M, Puchalski A (2008). Use of oromucosally administered interferon-alpha in the prevention and treatment of animal diseases. Pol J Vet Sci.

[24] Stuyven E, Cox E, Vancaeneghem S, Arnouts S, Deprez P, Goddeeris BM (2009). Effect of beta-glucans on an ETEC infection in piglets. Vet Immunol Immunopathol.

[25] Volman JJ, Ramakers JD, Plat J (2008). Dietary modulation of immune function by beta-glucans. Physiol Behav.

[26] Foster N, Berndt A, Lalmanach AC, Methner U, Pasquali P, Rychlik I (2012). Emergency and therapeutic vaccination—is stimulating innate immunity an option?. Res Vet Sci.

[27] Netea MG, Joosten LAB, Latz E, Mills KH, Natoli G, Stunnenberg HG (2016). Trained immunity: a program of innate immune memory in health and disease. Science.

[28] Jensen KJ, Benn CS, van Crevel R (2016). Unravelling the nature of non-specific effects of vaccines—a challenge for innate immunologists. Semin Immunol.

[29] Johansson E, Domeika K, Berg M, Alm GV, Fossum C (2003). Characterisation of porcine monocyte-derived dendritic cells according to their cytokine profile. Vet Immunol Immunopathol.

[30] Sjölund M, Fossum C, Martín de la Fuente AJ, Alava M, Juul-Madsen HR, Lampreave F (2011). Effects of different antimicrobial treatments on serum acute phase responses and leucocyte counts in pigs after a primary and a secondary challenge infection with *Actinobacillus pleuropneumoniae*. Vet Rec.

[31] Wikström FH, Fossum C, Fuxler L, Kruse R, Lövgren T (2011). Cytokine induction by immunostimulatory DNA in porcine PBMC is impaired by a hairpin forming sequence motif from the genome of Porcine circovirus type 2 (PCV2). Vet Immunol Immunopathol.

[32] Vandesompele J, De Preter K, Pattyn F, Poppe B, Van Roy N, De Paepe A (2002). Accurate normalization of real-time quantitative RT-PCR data by geometric averaging of multiple internal control genes. Genome Biol.

[33] Bálint A, Tenk M, Deim Z, Rasmussen TB, Uttenthal A, Csagola A (2009). Development of primer-probe energy transfer real-time PCR for the detection and quantification of Porcine circovirus type 2. Acta Vet Hung.

[34] Wallgren P, Persson M (2000). Relationship between the amounts of antibodies to *Actinobacillus pleuropneumoniae* serotype 2 detected in blood serum and in fluids collected from muscles of pigs. J Vet Med B Infect Vet Public Health.

[35] Sedlak C, Patzl M, Saalmüller A, Gerner W (2014). IL-12 and IL-18 induce interferon-γ production and de novo CD2 expression in porcine γδ T cells. Dev Comp Immunol.

[36] Stepanova H, Mensikova M, Chlebova K, Faldyna M (2012). CD4+ and γδTCR+ T lymphocytes are sources of interleukin-17 in swine. Cytokine.

[37] May L, van Bodegom D, Kuningas M, Meij JJ, de Craen AJM, Frölich M (2009). Performance of the whole-blood stimulation assay for assessing innate immune activation under field conditions. Cytokine.

[38] Peters SM, Yancy H, Bremer E, Monroe J, Paul D, Stubbs JT (2011). In vitro identification and verification of inflammatory biomarkers in swine. Vet Immunol Immunopathol.

[39] Magnusson M, Johansson E, Berg M, Eloranta ML, Fuxler L, Fossum C (2001). The plasmid pcDNA3 differentially induces production of interferon-alpha and interleukin-6 in cultures of porcine leukocytes. Vet Immunol Immunopathol.

[40] Wattrang E, Wallgren P, Fuxler L, Lindersson M, Fossum C (1997). Tissue chambers—a useful model for in vivo studies of cytokine production in the pig. Vet Immunol Immunopathol.

[41] Wilson NS, Yang B, Morelli AB, Koernig S, Yang A, Loeser S (2012). ISCOMATRIX vaccines mediate CD8+ T-cell cross-priming by a MyD88-dependent signaling pathway. Immunol Cell Biol.

[42] Salamano G, Mellia E, Candiani D, Ingravalle F, Bruno R, Ru G (2008). Changes in haptoglobin, C-reactive protein and pig-MAP during a housing period following long distance transport in swine. Vet J.

[43] Piñeiro M, Piñeiro C, Carpintero R, Morales J, Campbell FM, Eckersall PD (2007). Characterisation of the pig acute phase protein response to road transport. Vet J.

[44] Sautron V, Terenina E, Gress L, Lippi Y, Billon Y, Larzul C (2015). Time course of the response to ACTH in pig: biological and transcriptomic study. BMC Genomics.

[45] Martínez-Miró S, Tecles F, Ramón M, Escribano D, Hernández F, Madrid J (2016). Causes, consequences and biomarkers of stress in swine: an update. BMC Vet Res.

[46] De Buck M, Gouwy M, Wang JM, Van Snick J, Proost P, Struyf S (2016). The cytokine-serum amyloid A-chemokine network. Cytokine Growth Factor Rev.

[47] Alvarez B, Revilla C, Domenech N, Pérez C, Martínez P, Alonso F (2008). Expression of toll-like receptor 2 (TLR2) in porcine leukocyte subsets and tissues. Vet Res.

[48] Chaussabel D, Pascual V, Banchereau J (2010). Assessing the human immune system through blood transcriptomics. BMC Biol.

[49] Ghimire TR (2015). The mechanisms of action of vaccines containing aluminum adjuvants: an in vitro vs in vivo paradigm. Springerplus.

[50] Findlay L, Sharp G, Fox B, Ball C, Robinson CJ, Bird C (2011). Endothelial cells co-stimulate peripheral blood mononuclear cell responses to monoclonal antibody TGN1412 in culture. Cytokine.

[51] Darwich L, Mateu E (2012). Immunology of Porcine circovirus type 2 (PCV2). Virus Res.

[52] Oliveira S, Pijoan C (2004). Haemophilus parasuis: new trends on diagnosis, epidemiology and control. Vet Microbiol.

[53] Cray C, Zaias J, Altman NH (2009). Acute phase response in animals: a review. Comp Med.

[54] Nygard AB, Jørgensen CB, Cirera S, Fredholm M (2007). Selection of reference genes for gene expression studies in pig tissues using SYBR green qPCR. BMC Mol Biol.

[55] Feng X, Xiong Y, Qian H, Lei M, Xu D, Ren Z (2010). Selection of reference genes for gene expression studies in porcine skeletal muscle using SYBR green qPCR. J Biotechnol.

[56] Shirkey TW, Siggers RH, Goldade BG, Marshall JK, Drew MD, Laarveld B (2006). Effects of commensal bacteria on intestinal morphology and expression of proinflammatory cytokines in the gnotobiotic pig. Exp Biol Med.

[57] Duvigneau JC, Hartl RT, Groiss S, Gemeiner M (2005). Quantitative simultaneous multiplex real-time PCR for the detection of porcine cytokines. J Immunol Methods.

[58] McCulloch RS, Ashwell MS, O’Nan AT, Mente PL (2012). Identification of stable normalization genes for quantitative real-time PCR in porcine articular cartilage. J Anim Sci Biotechnol.

[59] von der Hardt K, Kandler MA, Fink L, Schoof E, Dötsch J, Brandenstein O (2004). High frequency oscillatory ventilation suppresses inflammatory response in lung tissue and microdissected alveolar macrophages in surfactant depleted piglets. Pediatr Res.

[60] Borca MV, Gudmundsdottir I, Fernandez-Sainz IJ, Holinka LG, Risatti GR (2008). Patterns of cellular gene expression in swine macrophages infected with highly virulent classical swine fever virus strain Brescia. Virus Res.

[61] Tang ZX, Chen GX, Liang MY, Rong J, Yao JP, Yang X (2014). Selective antegrade cerebral perfusion attenuating the TLR4/NF-kappaB pathway during deep hypothermia circulatory arrest in a pig model. Cardiology.

